# RIDB: A Dataset of fundus images for retina based person identification

**DOI:** 10.1016/j.dib.2020.106433

**Published:** 2020-10-20

**Authors:** M. Usman Akram, Anum Abdul Salam, Sajid Gul Khawaja, Syed Gul Hassan Naqvi, Shoab Ahmed Khan

**Affiliations:** a Department of Computer & Software Engineering, National University of Sciences and Technology Islamabad, Pakistan; b Department of Mechanical Engineering, National University of Sciences and Technology Islamabad, Pakistan

**Keywords:** Fundus imaging, Retinal imaging, Retinal identification, Biometrics

## Abstract

The paper describes a dataset, entitled Retina Identification Database (RIDB). The stated dataset contains Retinal fundus images acquired using Fundus imaging camera TOPCON-TRC 50 EX. The abovementioned dataset holds a significant position in retinal recognition and identification. Retinal recognition is considered as one of the reliable biometric recognition features. Biometric recognition has become an integral part of any organization's security department. Before biometrics, the information was secured through passwords, pin keys, etc. However, the fear of decryption and hacking retained. Biometric verification includes behavioural (voice, signature, gait), morphological (Fingerprint, face, palm print, retina) and biological (Odour, saliva, DNA) features [1]. Amongst all of them, retina based identification is considered as the spoof proof and most accurate identification system. Since the retina is embedded inside the eye thus is least affected by the outer environment and retain in its original state. Moreover, the vascular pattern in the retina is unique and remains unchanged during the entire life span. The data presented in the paper is composed of 100 retinal images of 20 individuals (5 images were captured from each patient). The dataset is supported by research work [2] and [7]. These research papers proposed retinal recognition algorithms for biometric verification and identification. The proposed method utilized both vascular and non-vascular features for identification and yields recognition rates of 100 % and 92.5% respectively.

## Specifications Table

**Table:** 

Subject	Retinal Image Analysis
Specific subject area	Human retina, Biometrics
Type of data	Images [10]
How data were acquired	Images were acquired using TOPCON-TRC 50 EX Fundus Camera
Data format	Raw images [10] JPEG (.jpg) 1504 × 1000 Images Image Nomenclature is IM00000X_Y where Y represents person and X represents image number for a specific person
Parameters for data collection	Macula Centred Fundus Images with 45° Field of View (FOV)
Description of data collection	The dataset contains fundus images of the human eye which helps in analysing and examining the inside of an eye. The dataset mainly focuses on the biometric application of fundus images for retina recognition and identification.
Data source location	The data was collected from the Armed Forces Institute of Ophthalmology (AFIO). Rawalpindi, Pakistan 33.5962° N, 73.0450° E
Data accessibility	Repository name: Mendeley Data[10] Data identification number: 10.17632/tjw3zwntv6.1 Direct URL to data: https://data.mendeley.com/datasets/tjw3zwntv6/1
Related research article	Waheed, Z., Akram, M. U., Waheed, A., Khan, M. A., Shaukat, A., & Ishaq, M. (2016). Person identification using vascular and non-vascular retinal features. *Computers & Electrical Engineering*, *53*, 359-371 [2]. https://doi.org/10.1016/j.compeleceng.2016.03.010

## Value of the Data

• RIDB is comprised of retinal fundus images, useful to extract the retinal features which play a vital role in retinal identification i.e. Biometric verification/identification.
• The abovementioned database is significant in retinal feature analysis, which may help researchers in carrying out research based on the retinal biometric system verification and improvement.
• The Stated dataset is of significant importance since it's comprised of retinal images acquired from the left and right eye of each of the 20 individuals, 5 samples each. Multiple samples from each individual will ensure accurate capturing of retinal features which will aid in testing/training of biometric systems playing a beneficial role in the security system of organizations.

## 1 Data Description

Biometric features are amongst one of the significant features in an individual's identification. Amongst all biometric features i.e. face, iris, palm, retina, hand and palm geometry, signature, and voice, human retina is the best attribute to consider [3] for identification and verification; as it ensures unique vascular pattern resulting in accurate identification with the least probability of spoofing as it's embedded inside a body organ [4] and hardly changes over time [5]. Templates used for retina recognition and matching are quite small in size which makes the recognition and matching procedure quick [6]. Therefore, retinal images contained by the stated dataset plays a significant role as a data source in biometric identification and verification algorithms based on retinal features.

**Fig. 1:**
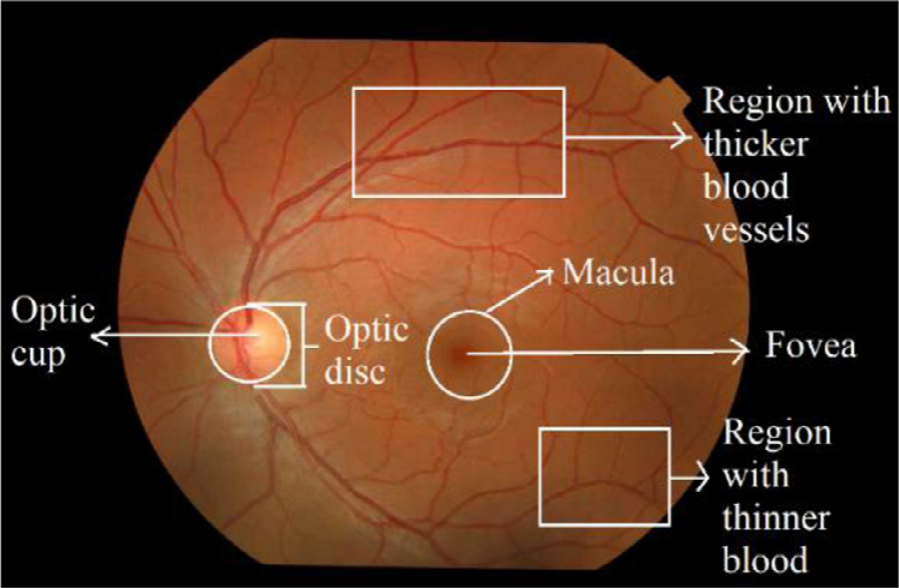
Fundus image Sample from RIDB - Inside of Human Eye.

Fundus imaging has been used to capture inside of the human eye i.e. retinal layers. Besides identification of retinal anomalies such as variation in Colour saturation, Haemorrhages, exudates, and lesions which may lead to optical ailment, retinal images acquired using Fundoscope may also aid in the identification of retinal features which aids in biometric-based identification and may be used to discriminate eye retina. Upon anatomization of the retina, it has been observed that eye retina is composed of components like optic cup, optic disc, fovea, macula, and blood vessels. The above-stated components are a rich combination of both textural and intensity-based features. Fig. 1 depicts the variation of contrast that can be seen from the cup region (the most lighted region) to the fovea (the darkest region) in the fundus image [1]. Similarly, the region containing an intense vascular map differs in textural properties compared to the non-vascular region. In addition to that, vascular structure (orientation and magnitude) along with its pattern also varies from an individual to another. Such retinal features, act beneficiary in analysing and identifying individuals.

Retina Identification Database (RIDB) is a retinal image database comprised of 100 Fundus Images with 1504 × 1000 resolution compressed in JPEG format. These images are captured using TOPCON-TRC Fundoscope, from 20 individuals (five samples per person) having no retinal disease. Therefore, the dataset contains healthy retinal images with no signs of retinal diseases and anomalies. The supported research [2] is based on biometric identification using both vascular retinal features i.e. blood vessel orientation and angle, along with non-vascular retinal features i.e. luminance, contrast, and structure. The coupling of these features resulted in a more robust retinal recognition system.

**Fig. 2:**
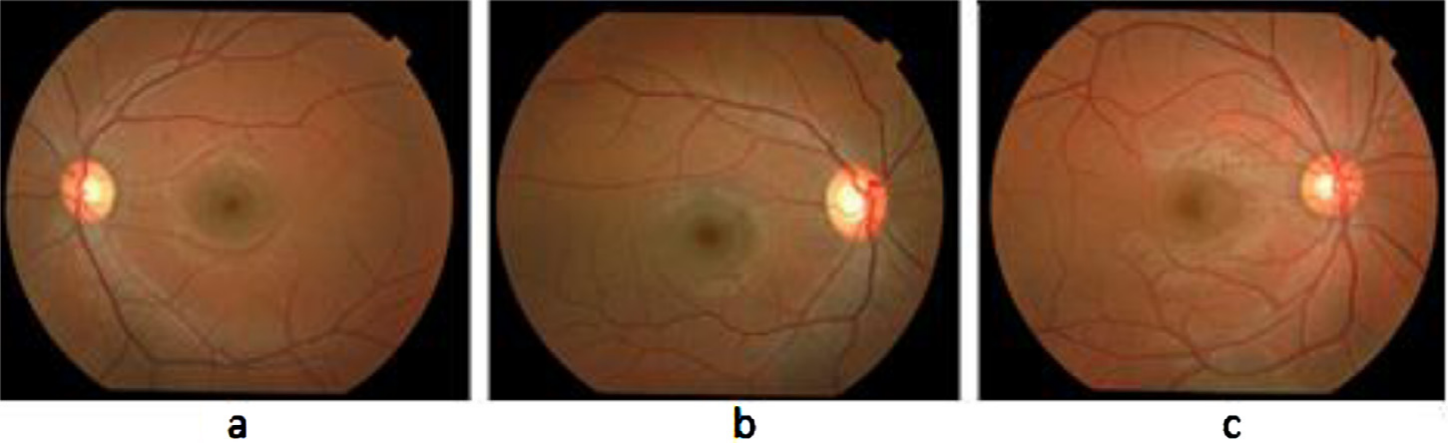
Some Fundus Images samples from RIDB.

Fig. 2 shows some images of the left and right eye of different individuals contained by RIDB. Keen observation of these Images acquired from different individuals revealed the fact that all of them vary in blood vessel orientation, intensity, and magnitude. Moreover, the color and textural based features may also vary from a person's retina to another which adds uniqueness and distinction to the feature vector. Therefore, retinal images hold a significant value in analyzing retinal features which helps in retinal identification of an object to another.

## 2 Experimental Design, Materials and Methods

The fundus imaging camera for Angiography with model number TOPCON-TRC 50 EX was engaged to capture the retinal images. The above-stated fundus camera is being used to study lesions of vitreous, choroid, and retina through infrared or fluorescein angiography. Image acquisition was preceded by pupil dilation Ø4.0 mm and with 45o FOV. Each image is captured with a spatial resolution of 1504 × 1000 and saved in JPEG format.

**Fig. 3:**
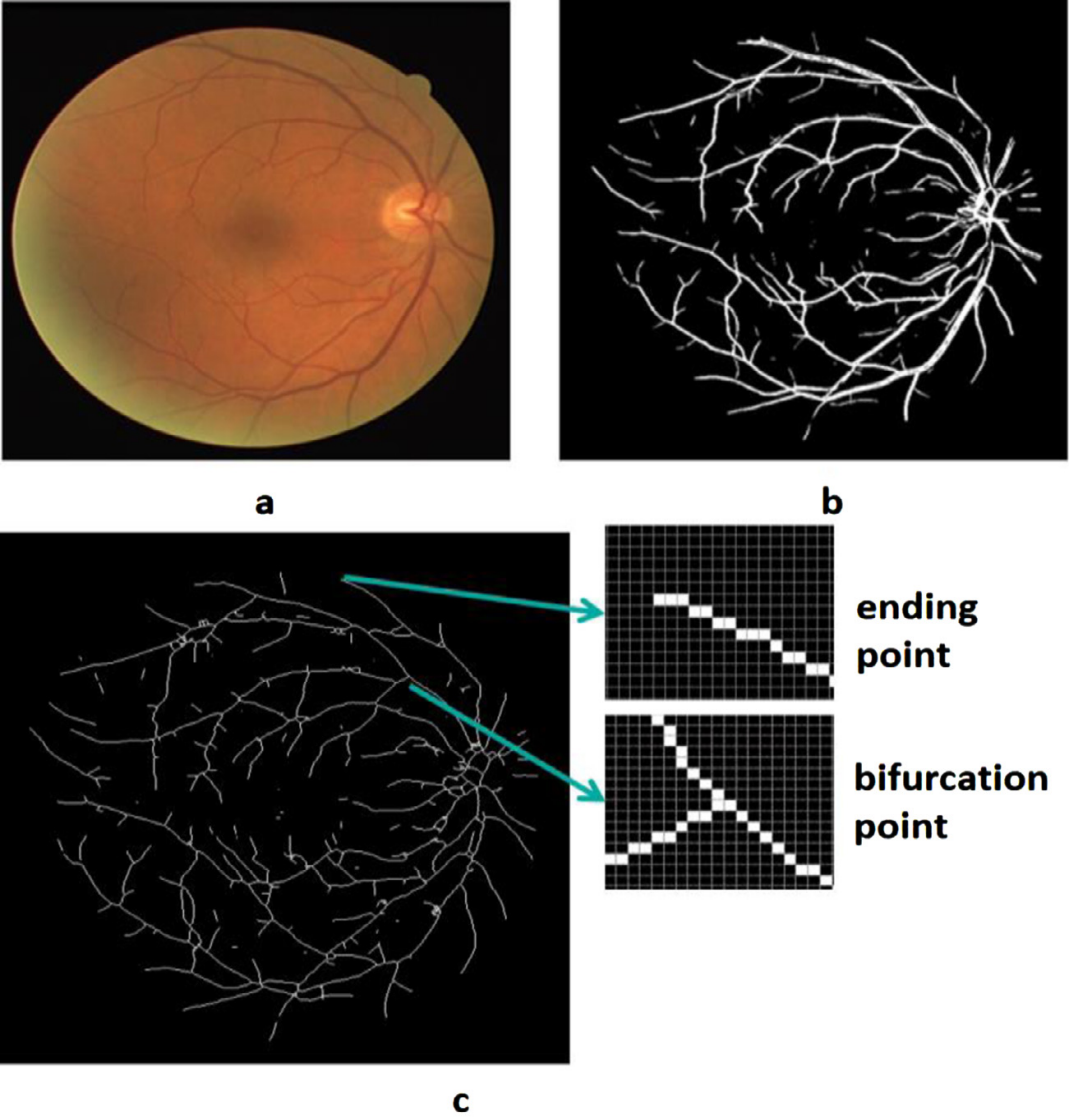
(a) Fundus image (b) Extracted Vascular Map (c) Vascular Features I.e. Ending point, bifurcation point.

The data set has been used for retina recognition and identification systems presented in [2] and [7]. The proposed retinal identification system is a more robust algorithm as it used both vascular and non-vascular features for retinal recognition. Vascular based feature extraction in the presented research is preceded by accurate blood vessel extraction. Accurate blood vessel extraction is quite an exigent task due to the presence of haemorrhages, exudates, lesions, and micro-aneurysms which may be categorized as false vessels, thus affecting the accuracy of results [8]. Therefore, an improved blood vessel extraction algorithm was proposed proceeded by minutiae points identification, followed by feature extraction. Retinal vascular based features include bifurcation (split of a vessel into two), branch (origin of small sub vessel from a major vessel), crossover (crossing of two vessels), and ending (ending point of a vessel) [9]. These features are a crucial factor in retinal based identification since unique vascular map exists from an individual to another. Amongst these vascular features, research work proposed in [2] and [7] identified bifurcations and endings of vascular patterns as Minutiae points for feature extraction. Fig. 3 shows the vascular map extracted from RIDB Fundus image, followed by minutiae points (bifurcations, endings) extraction. Against each extracted feature point, four nearest neighbour features are utilized to generate a 1 × 8 rotation-translation invariant feature vector comprised of the relative angle of of F_i_ and F_j_ i.e. dij and relative Euclidean distances dij, <Ĉ_f1f2_ Ĉ_f1f3_ Ĉ_f1f4_ Ĉ_f1f5_ d_f1f2_ d_f1f3_ d_f1f4_ d_f1f5_>_,_ used for training and testing the system. Cosine similarity index was used as a scale for calculating the similarity.

The second module of the research used non-vascular features such as Luminance, contrast, and structure for retinal recognition. Experimental evaluation was conducted on 4 datasets including RIDB. Recognition rates of 100% and 92.5% were achieved using vascular based features and non-vascular based features respectively.

## Ethics Statement

All examination procedure was conducted in accordance with the instructions of the ophthalmologist. Before capturing images, the consent of patient has been taken for being included in the research. Keeping in view the hospital's private-policy regarding patient data, the data does not contain any information regarding the patient's name, age, address details.

## Credit Author Statement

**M. Usman Akram:** Conceptualization, Methodology, Software, Supervision, Project Administration. **Anum Abdul Salam:** Writing-Original draft, review & editing**. Sajid Gul Khawaja:** Data curation, Visualization. **Syed Gul Hassan Naqvi:** Investigation, resources**. Shoab Ahmed Khan:** Validation, Formal Analysis, Funding acquisition.

## Declaration of Competing Interest

All authors declare that they have no known competing financial interests or personal relationships which have, or could be perceived to have, influenced the work reported in this article.
